# Potential and limitations of digital ethnographic research: A case study on a web community

**DOI:** 10.3389/fsoc.2022.1092181

**Published:** 2023-01-04

**Authors:** Giuseppe Masullo, Marianna Coppola

**Affiliations:** ^1^Department of Human Sciences, Philosophy and Education, University of Salerno, Fisciano, Italy; ^2^Department of Political and Communication Sciences, University of Salerno, Fisciano, Italy

**Keywords:** digital ethnographic research, case study, typology of asexual people, shortfalls and advantages, qualitative research

## Abstract

**Introduction:**

This work aims at transposing ethnographic research into digital contexts to probe its potential and limitations in a specific field of study: that of sexuality, particularly suited to ethnographic exploration. We chose as our case study a web community of Italian asexual people. As we shall see, this allowed us to simultaneously explore both the various techniques called into play in digital ethnography and the digital as a specific sphere within which sexuality takes on a very peculiar meaning. Digital sociality is paramount for the definition of imaginaries, meanings, and practices that could not be explored elsewhere. This is due to the implicit characteristics of the population studied, which does not find corresponding physical spaces of aggregation.

**Methods:**

The paper will present the research design using this specific case study to address some of the typical dilemmas that researchers face when following the digital ethnographic approach and will explore the research results as an example of the kind of analysis available with the information and data collected through this method.

**Results and discussion:**

The conclusions will attempt to briefly outline the shortfalls and advantages of this method, considering its application to this specific field of study.

## Introduction

Among the social research methods, ethnography is one of the most comprehensive tools available to researchers to reconstruct the visions, perspectives, imaginaries, beliefs, values, and practices that underpin a given culture (Masullo et al., 2020). It is no coincidence that many manuals on social research methods and techniques consider the ethnographic approach to be among the most representative of a “specific” way of doing research. Ethnography also has the merit of successfully combining three procedures that may not simultaneously come into play in research inspired by the interpretative tradition, namely: observing, questioning, and reading (Corbetta, 2005). In ethnographic research, researchers immerse themselves fully in their field of research—and, in some cases, are themselves part of it as members of the community investigated (auto-ethnography). In this type of study, all the senses are put to the test by the objective of the investigation. The choice of such an approach is not neutral and implies upstream decisions that are articulated along three planes: ontological, epistemological, and methodological. The ontological one refers to a reality understood as a social construction of meaning. The epistemological relates to the relationship between researchers and the object of the research. Finally, the methodological one concerns a multi-method approach involving the use of different techniques of information-gathering: observing (participant observation), questioning (the interview), and reading (triangulation with secondary data sources).

This work aims at transposing ethnographic research into digital contexts to probe its potential and limitations in a specific field of study: that of sexuality, particularly suited to ethnographic exploration (Delli Paoli, 2022). We chose as our case study a web community of Italian asexual people. As we shall see, this allowed us to simultaneously explore both the various techniques called into play in digital ethnography and the digital as a specific sphere within which sexuality takes on a very peculiar meaning. Digital sociality is paramount for the definition of imaginaries, meanings, and practices that could not be explored elsewhere. This is due to the implicit characteristics of the population studied, which does not find corresponding physical spaces of aggregation.

The first section of the paper will detail some theoretical aspects relating to the digital ethnography approach, analyzing the similarities and differences with the traditional ethnographic approach, followed by a description of the community under investigation (asexual people). We will highlight the processes that lead this sexual minority to consider the digital environment as the only place in which to self-define and express themselves. The second section of the paper will present the research design using this specific case study to address some of the typical dilemmas that researchers face when following the digital ethnographic approach. The third section will explore the research results as an example of the kind of analysis available with the information and data collected through this method. The conclusions will attempt to briefly outline the shortfalls and advantages of this method, considering its application to this specific field of study.

### The digital ethnographic approach: Similarities and differences with the traditional ethnographic approach

Born in recent years as a transposition of the classical ethnographic approach into the digital environment, digital ethnography is in some respects still an unexplored field currently attracting young as well as more experienced scholars (Masullo, 2020). Its transversal pull can be ascribed to the fact that, though retaining many of its original features (directly linked to the hermeneutic sociological tradition), its application to the digital environment and the interpretative and methodological challenges it entails bring out new potential. The methodological literature on this approach is not yet systematic, as shown by the plurality of terminological labels attributed to it. Some of these, mainly used in the sociological field, frame this approach in the tradition of digital sociology and digital methods and thus speak of “digital ethnography” (Murthy, 2008); others, particularly in marketing, refer to the importance of the network and thus define it as “netnography” (Kozinets, 2002, 2010, 2015)^1^.

The new information and communication technologies greatly affect many areas of people's life and, therefore, many processes at the heart of the sociological investigation. Focusing on micro-sociological aspects and everyday life, platforms and new communication tools have engendered *for* and *in* individuals a new way of conceiving themselves and their reality. They broadened their social and collective horizons and their way of meeting and interacting with others (Masullo and Addeo, 2021). The pervasiveness of technology and the ubiquity determined by the so-called “internet of things” configure new realities in which some juxtapositions are irrelevant and no longer explicative—for example, online/offline, virtual/real, material/immaterial (Garcia et al., 2009; Beneito-Montagut, 2011; Scaramuzzino, 2012). The normative and value references connected to social action no longer relate only to a precise sphere delimited in space and time but expand through the subjects' ability to surf the net and take full advantage of all the potential (informative, communicative, and relational) offered therein. In the face of the current expansion of the web society, the perspectives of ethnographic research are expanding in tandem with the digital world. Concerning the objects of study, we can distinguish between the exploration of classic sociological objects of study and how they can be rethought in the digital sphere and through digitisation, or the exploration of natively digital phenomena, which arise directly within the web. In the latter case, the web becomes both the field in which the observation takes place and the context in which the phenomenon itself originated. Initially, the ethnographic approach applied mainly to online communities, delimited digital spaces of social aggregation around a specific domain of interest. In recent years, however, these privileged sites have been supplemented or sometimes replaced by social media sites and metadata in digital ethnographic research. The ongoing rise of these new spaces for ethnographic fieldwork, in turn, promotes new types of ethnographic practice that are still partly unexplored (Delli Paoli, 2022). Despite this change, ethnographic activity retains its original meaning, namely the interest in culture as a text that must be decoded by the ethnographers, who cannot merely read the data. It is, therefore, still assumed as ontologically central that creation and creativity are inherent in ethnographic research (Delli Paoli, 2022). Geertz (1973) defines “thick descriptions” as deep cultural representations, which do not stop at the exteriority of things, but take into account stratified cultural meanings and thus manage to unravel the fabric of culture and produce descriptions that are consistent with the indigenous point of view. On an epistemological level, the process of cultural translation appears (including in the digital environment) as a tension between foreignness and familiarity, in the dialogical dialectic between detachment and empathy. Just as in in-presence ethnography, digital ethnographers must maintain what Davis (1973) defines as the balance between the Martian, who strives to maintain detachment from the cultural and cognitive assumptions of the natives, and the convert, who identifies totally with the cultural models of the natives. On the methodological level, digital ethnography reaffirms the centrality of observation. As in classical ethnography, such observation can involve a different involvement of the researcher in the community under investigation. However, unlike classical ethnography, this observation opens up further, hidden forms of observation that are not possible in the case of the physical participation of the researcher on the field—as it is not possible to conceal his presence. The literature is divided between proponents of the two types of observation. The arguments in favor of overt observation revolve around the ethical and deontological aspects of research and the need to reveal to the subjects that they are being observed and studied. From this point of view, covert observation, also known as covert access or lurking, would be an unethical practice. Arguments in favor of covert (or lurking) observation, on the other hand, emphasize the non-intrusiveness of this method, which favors the “naturalness” of the information gathered (Masullo et al., 2020).

From this point of view, Delli Paoli (2022, p. 200) observes that “On the one hand, there are scholars who suggest that lurking is not an ethnographic observation in the traditional sense and therefore not a “correct” ethnography (....) it provides any deep understanding of the community, but only a superficial description. On the other hand, there are scholars who idealize the possibility of lurking, which would offer a unique opportunity for “natural” data collection, as members are unaware of their status as informants and the presence of the researcher does not cause them to change their behavior”.

The choice between one and the other type of observation is not completely free. In cases where the presence of a researcher would not be welcome, for instance in the case of sexual minorities or practices at the limits of legality, covert observation remains the only possible way into the field.

Other specificities of the digital ethnographic approach, compared to classical face-to-face ethnography, are to be found in its efficiency in data collection, which requires much less time, and its opportunity to expand the geographical dimension of the research field and connect networks scattered all over the world. The researcher does not need to travel anywhere; information can be located and stored on the Internet without having to be recorded and transcribed as the traditional ethnographer must do (Kozinets, 2002). Another strength is the invisibility and relative discretion of the researcher: cyberspace allows researchers to be invisible to the people they are observing more easily than in face-to-face observation (Kozinets, 2010; Scaramuzzino, 2012; Murthy, 2013; Varis, 2014; Masullo et al., 2020). However, the digital ethnographic approach also has some limitations compared to in-person research. In the online environment, the episodic nature of the relationship that the members have with a virtual community (such as a blog, a discussion forum, or a Facebook group) requires a rethinking of the concept of community and communitarianism and makes it difficult to investigate relevant aspects in physical contexts, such as those of a structural nature relating to the dimension of power (which in the sociological sense cannot be deduced only from the level of participation of the users, nor from the configuration of the posts, opinion leaders, and shifts in interaction). The level of involvement of the researchers in the community studied will also vary depending on the degree of familiarity they can create with the members of a community—who, moreover, are ever-changing and for whom socio-demographic characteristics (gender, age, ethnicity, educational qualification, social class, etc.) do not always come to the fore (or are not always true in the digital sphere). The latter aspect makes it clear that digital ethnography cannot be considered a mere transposition of physical ethnography, and that the renunciations it requires are acceptable in the case of phenomena that find their only form of expression in the digital world and require a multi-method approach of exploration (of observing, questioning, and reading).

The following section will describe in detail the phenomenon of asexuality to provide some characteristics of the population that recognizes itself in this expression of sexual orientation. This will also allow us to grasp the reasons why digital ethnography is considered a particularly valid approach for studying hidden populations, which find many spaces for their expression in the digital environment (Monaco, 2021).

### The phenomenon of asexuality: A literature review

In recent years, the number of self-described “asexual” people has increased. In the literature, asexuality (or the acronym ACE) is defined as a sexual orientation in which the person declares an absence and/or a consistent reduction in sexual and erotic attraction or frequency of face-to-face sexual practices (Decker, 2015; Gupta, 2017). Recently, the definition has been updated in the experience of little or no sexual attraction to include a more comprehensive spectrum of experiences of sexual attraction (Carrigan, 2011; Decker, 2015).

The study of asexuality has prompted the scientific community to trace the possible motivations and explanations that contributed to the formation and spread of the phenomenon. On the other hand, it also made explicit the need to rework and reconsider the normative parameters on the meaning of sexuality, which in our society is often understood as a *sine qua non* of romantic and emotional relationships between partners. In a context characterized by a widespread appeal to sexuality, asexuality challenges the dominant conceptualization of sex as a universal and natural dimension and sheds light on the different ways in which individuals conceive it depending on their biographical experience and subjective desires (Delli Paoli and Masullo, 2022).

Bogaert and Skorska (2011), one of the main authors who studied asexuality, highlights two different subcategories: *primary*, in which the subjects never experienced a hetero-referenced sexual attraction/interest, and *acquired*, in which the subjects, after a period of hetero-referenced sexual attraction/interest, define themselves as ACE due to personal and social motivations that scholars are beginning to explore.

A further distinction (and sub-classification) within the ACE condition stems from the studies of Poston and Baumle (2010), who highlighted how some categories of people cannot be included in that of asexuality. For example, those who choose chastity before marriage, or who are celibate for religious reasons, or, finally, INCELS (involuntary celibates), subjects in whom sexual attraction and erotic desire are not absent, but “unexpressed” due to social, psychological, or cultural conditions.

Lehmiller and Gormezano (2022) pointed out that asexuality affects about 1% of the American population, pinpointing four aspects that identify this condition.

a) *It does not correspond to chastity*. Both conditions are characterized by the absence of sexual activity, but their motivations differ. Asexual people experience a total or partial absence of erotic desire and sexual attraction toward others. Conversely, people who choose chastity continue to have sexual attraction toward other people.
b) *It is not a sexual dysfunction*. The asexual condition is a normal and possible expression of sexual orientation; it is not related to any organic or psychological pathology of the sexual sphere.
c) *It is not related to inexperience*. Asexuality is not attributable to shyness or other expressions of a psychological nature.
d) *It is not devoid of autoeroticism*. While it is true that asexual persons are not attracted to other people, this does not imply that they avoid regular autoeroticism and sexual self-gratification.

For Lehmiller, the asexual condition is ultimately an identifiable and well-structured sexual orientation in its own right.

The scholar also proposes a further classification of asexual persons depending on their relationship with sex and sexual practices, distinguishing between the following: (a) *sex-repulsed*, i.e., people who feel repulsion toward sex or some specific elements of it; (b) *sex-averse*, i.e., people who have no intention of having sexual experiences, who are distinguished from sex-repulsed in that a sex-averse person does not necessarily feel repulsion toward general sex, but does not consider it a central aspect of their existence; (c) *sex-indifferent*, those who do not have a particular interest in sex; (d) *sex-favorable*, people who experience interest and desire in sex, without being reflected in a constant search for sexual experience.

The asexual condition does not exclude sentimental and romantic attraction to other people, an emotional attraction that is not reflected in a sexual experience (the latter being understood as a practice). Asexual persons, therefore, can be identified as *homoromantic*, who experience emotional attraction to persons of the same sex, *heteroromantic*, who experience relational and emotional attraction to persons of the opposite sex, *biromantic*, who experience emotional attraction to both sexes, *panromantic*, who experience attraction to other people regardless of their sex and gender identity, and, finally, *aromantic* (AroAce in the literature) who do not experience sexual, emotional, or relational attraction to any person, regardless of their gender and sexuality.

The asexual condition has been on the rise in recent years among adolescents and young adults, to the point that some scholars are questioning whether this constitutes a generational trait of our age which needs to be addressed. To test this hypothesis, McInroy et al. (2021) recently conducted a study of 600 Americans aged between 14 and 24 in which they found that around 24% defined themselves as not interested in sex or sexual practices, a percentage that almost doubles in the 14 to 18 years old cohort, to around 45%. The authors link this condition, particularly for younger people, to a phase of “identity instability” or a “transitional” phase of self-knowledge, a hypothesis also supported by the progressive reduction in the percentage of asexual people as age increases.

Studies show that the condition of asexuality and aromanticism is stigmatized not only within mainstream society but also in the LGBTQ+ environment, as it is considered unnatural and/or related to dysfunctional aspects of the psychological or sexual sphere (Robbins et al., 2016).

In this regard, a study conducted by MacNeela and Murphy (2015) on an LGBTQ+ online community found that around 56% of members had not made their asexual orientation explicit in their profile presentations, and that disclosure of their asexual status only occurred at a later stage and/or during an offline meeting.

Finally, Gupta (2017) traces similarities and differences between the coming out of other non-conforming identities in the LGBTQ+ community and that of asexual and aromantic people. In both cases, there is a desire to come out of the closet, for authenticity and the possibility to relate to people coherently, explicitly highlighting fundamental aspects of the process of identity self-determination. However, this choice also increases the subjects' vulnerability to negative experiences such as harassment, discrimination, marginalization, and violence, since in mainstream society the absence of sexuality is not accepted and integrated—where sexuality is understood as an obligation to which both men and women (albeit with different meanings) are naturally called upon to respond (Kosciw et al., 2013; Gupta, 2017).

Recent studies in Italy have confirmed the preference of LGBTQ+ people for digital environments to make their sexual identity explicit and as a specific field of socialization to sexuality, also considering the persistence of a general homophobic and transphobic culture together with the repudiation of alternative sexual expressions. Consequently, the latter enjoy a greater possibility of being experienced in digital spaces than in offline reality (Carrigan, 2011; Bacio and Peruzzi, 2017; Masullo and Coppola, 2020, 2022). Although the condition of asexuality constitutes a sexual orientation in its own right, it shares some characteristics with other subjectivities of the LGBTQ+ universe: it is, to all intents and purposes, one of the “non-normative”' sexual orientations and, therefore, contrast with the imposition of a prevailing sexual model, which finds its *raison d'être* in “reproduction”. Furthermore, it experiences the same mechanisms of discrimination insofar as this orientation does not find space for its open and complete explicitness in the environments of public society.

The recent digital revolution has affected various spheres of everyday life, broadening and complexifying the social and communicative contexts and spaces for everyone.

The creation of “virtual spaces” has represented a precious opportunity for social emancipation for those subcultures that previously struggled to find aggregative contexts and opportunities for confrontation in mainstream and offline society.

These resources for “emancipation” have proved to be suitable and convenient for the LGBTQ community and asexual people who, thanks to the peculiar characteristics of the web society, have created different and diversified tools for knowledge, comparison, aggregation, and the search for possible sentimental and/or sexual partners.

Cyberspace represents the main, if not the only, space for the aggregation and sharing of opinions, reflections, and of identity confrontation for the new non-conforming sexual identities, such as non-binary, pansexual, and asexual people.

On the one hand, these “new instances” find in the virtual sphere impulses and identity drives to emancipate themselves and consolidate their process of self-determination. On the other hand, however, it is precisely within the online community that they experience forms of discrimination and social disavowal.

In this regard, Smith (2012) spoke of the *delegitimisation of the ACE identity* from public discourse, defining it as an *invisible and denied society* in the offline and online mainstream community, positing the need for specifically dedicated, private, closed, and selective communicative spaces and spheres of confrontation.

The need to build spaces of emancipation and sharing specifically for ACE persons has given rise to the creation of numerous communities, chats, and social pages for this condition worldwide.

McInroy et al. (2021) recently investigated the use of online communities by people who self-define as asexual and aromantic, highlighting certain functions considered fundamental to a process of *self-determination* and *self-definition*. According to the research data, about 14.6% of the participants stated that they had attended or were attending online LGBTQ+ support groups to find information, to find information and *clarifications* about their condition for the self-determination process. Another important aspect is the search for information on pathologising the Ace condition: about 45.7% of participants sought and requested news, information, and experiences on mental health, psychological, biological, and sexual aspects possibly involved. Finally, 34.5% of participants explicitly stated a relational and social purpose of using the community, highlighting how expressing oneself in a safe, albeit virtual, place is among the main motivations to join the platform, as well as to look for people with similar or partly overlapping experiences.

## Materials and methods

### Research design

Starting from the theoretical premises argued above, this section aims to document the various steps of digital ethnographic research in the light of the specific field examined here, that is, the processes of self-definition of the users of the Italian online community dedicated to asexual people, to identify and analyse common traits and differentiations in the imagery and use of cyberspace. It should be noted that this essay follows previous work on the AVEN (Asexual Visibility and Education Network) web community, one of the most important online communities of asexual people, aimed at analyzing the processes of self-identification, as well as the plurality of experiences and attitudes expressed by these people in their request for greater freedom from the constraints of sexuality as a necessary imperative for building meaningful relationships with others, including on a romantic level (Delli Paoli and Masullo, 2022).

We decided to replicate the same study in a community frequented mainly by Italian people, given that the previous research focused on people familiar with the English language. This choice excluded those who did not speak the language and generated a partial view of the phenomenon in the country. While it is true that patriarchy and heterosexism almost universally shape how relate to the identity-related dimensions of gender and sexuality, in Italy these normative axes can affect them in a very peculiar way. Therefore, we decided to apply the same research approach (that of digital ethnography) to explore in greater detail this specific Italian web community in which relational and power dynamics may be at work in a different way from the case previously examined^2^.

The first fundamental step in the ethnographic research was the definition of the field which, as Kozinets (2015) points out, concerns not so much the characteristics of the medium or its use, but rather the cultural, relational, and value experiences developed within cross-media digital spaces—in other words, the digital worlds of meaning.

As already out above, digital ethnography was initially born to study online communities that organized themselves around shared lifestyles, values and moral beliefs, emotions, and consumption practices (Cova, 1997). The recent technological developments of Web 2.0 and the pervasive diffusion of mobile devices forced the research to adapt to the fact that the spaces and times of online discussions have become increasingly transmedia and linked to a thematic domain rather than a single medium. As Delli Paoli points out, “Most online interactions take shape in a volatile context, without defined spaces but with content delimited by the use of tags, algorithms and data mining techniques that organize the flow of information and act as transversal metadata across web pages, allowing actors to move in non-linear directions from one medium to another” (Cova, 1997, p. 46).

Given its digital nature, the netnographic approach cannot be media-centric—i.e., tied exclusively to the study of defined online spaces such as blogs and communities.

Adopting the distinction between meta-fields as spaces unrelated to a media and built around a topic and contextual fields as contextualized spaces in blogs, communities, discussion forums, social media groups, etc. (Airoldi, 2018; Delli Paoli, 2022), the study opts for the latter by examining the Italian online community, which has around 3,000 users. The cases observed are the result of a reasoned selection based on “theoretical sampling” criteria that envisage the selection of typical cases able to provide the best opportunities to find the information necessary for the study and that, as a sample, can be close enough to the population analyzed (though not representative).

Digital ethnography can be considered a distinctive method to study social changes resulting from the digital world itself. In the case of sexuality, for instance, the digital sphere offers unprecedented discursive spaces to those sexual minorities that find no space in offline reality. It is in the digital world that these minorities find the full possibility for self-expression (as in the case of asexual people) and within this context that these individuals interact and construct their own language, giving rise to specific practices and scripts that would not be possible or imaginable outside this sphere (Rinaldi, 2016; Delli Paoli and Masullo, 2022). Digital ethnography thus proves to be particularly appropriate as a research approach, especially to study those phenomena born in the digital realm, and to investigate generative and productive (and not only reflexive) digital identities and cultures, making it possible to document the performative use of language (Butler, 2004).

The second step entailed the definition of research questions. From this point of view, the digital ethnographic approach highlights the typical advantages of interpretive approaches, insofar as there is no sequential order between field definition and research questions (Hammersley, 1995). While it is true that in some cases the research questions guide the selection of the field, the difficulty of finding information on the asexual condition, which is considered a hidden and invisible population in offline reality, determined the need to first select the field of study, and then the research questions. Nevertheless, the latter also gradually emerged during the observation, given that this is a virtually unexplored field in Italian research.

The lack of studies on the subject does not imply that the research approach lacks a theoretical foundation. On the contrary, we believe that the choice of field, and the selection of what to observe, are choices that must always be contextualized to the fields and objects examined (in our case, that of sexuality). It could not be otherwise: an observation without a guide, not oriented by what Blumer (1954) called “sensitizing” concepts, would prevent the researchers from selecting and distinguishing, within the reality observed, the “meaningful” elements from those “banal” and misleading. Moreover, observation can only take place based on a series of pre-cognitions relating to the field one intends to explore. As we can learn from one of the most famous community studies, conducted by Lynd and Lynd (1970) on Middletown, observation must always be preceded by background research that includes not only the study of specific literature on the subject but also documentary analysis (in the case in point, the study of statistical sources).

Another fundamental aspect was the accessibility of the field. In this case, it depends on both the characteristics of the platform being examined, i.e., its “accessibility” and the need to choose an online community in which the level of interaction is particularly intense and which for the chosen topic constitutes a reference in the digital environment. Regarding the first point, the asexual community allows access only after registering and filling in a profile. Regarding the second point, the chosen online community represents the main space where Italian asexuals meet. This digital field was chosen after an exploratory observation aimed at detecting the main users and gatekeepers, the intensity of interactions and the affordances of the platform.

From the outset, the research was confronted with ethical dilemmas, directly related to its objectives and the techniques it intended to employ. Regarding observation—understood as the main technique of digital ethnographic research—we opted for a mixed mode between covert access and the explication of our identity as researchers. From an ethical point of view, as pointed out above, the omission of identity becomes justifiable and legitimate, as some scholars claim, in certain circumstances, especially when the benefits outweigh the social and ethical costs of such a violation. In this case, for instance, making the researcher's role explicit from the outset would have made the field inaccessible.

In the first phase, we gathered information covertly without revealing our presence to those concerned, a more appropriate—if ethically questionable—choice for studying invisible populations. In the second phase, we informed users of the research and our role as researchers. We never intervened to alter the context of the interactions. On the contrary, we strove to preserve the ecology of the environment and, therefore, our method can be defined as non-participant observation.

The observation period went from 22 October to 22 December 2021. We examined 200 profiles and presentations and over 500 related posts, collected in a specific excel grid. Alongside the grid, we drew up a daily diary in which we noted down field notes related to what we read in the online community, which proved valuable in the definition of the first research questions. Among the most significant, which guided the subsequent steps of the research, were the following:

RQ1: What are the main motivations and/or paths that lead the individual to choose a relational modality involving the absence and/or reduction of sexual activity?
RQ2: How do users use the community and for what purposes?
RQ3: How do users define themselves in relation to the different meanings attributed to the concept of asexuality?
RQ4: What differences emerge between the way users define themselves and their main socio-demographic characteristics?

The researchers' identity was later made explicit by contacting certain users willing to answer questions through a private messaging system provided by the platform. This procedure constitutes the second technique employed in this study: we decided not to limit ourselves to “observing” but also to “question” our cases, for two main purposes: (1) to clarify certain meanings connected to the language typical of this subculture which could have escaped the researcher inexperienced in the universe examined, (2) to delve deeper and reinforce certain intuitions gathered during the observation phase.

About the “reading”, we decided to examine 200 profiles in the observed period. We proceeded to extrapolate a series of information on socio-biographical variables to infer possible associations between them and certain traits of asexual persons identified in the literature.

The decision to analyse the ecological information made available by the medium Rogers (2013) when he affirmed the *follow-the-medium* principle as foundational to digital methods: the researcher is called upon to follow the ontological properties of the medium, to immerse himself in it, to equip himself with a methodological apparatus that is natively digital by making the technical strategies and natural logics of digital media his own and using them as methodological sources.

For clarity's sake, we described the operations of “observing”, “questioning”, and “reading” sequentially, in relation to the three techniques employed in this study. However, they most often occurred in parallel, taking full advantage of the flexibility of the ethnographic approach.

The last phase of the research concerned information analysis and was mainly conducted through qualitative content analysis approaches, also known as Ethnographic Content Analysis (Altheide, 1987).

Content analysis is essentially based on the interpretation and classification of texts with the help of the most diverse, sometimes competing, and contradictory procedures (Rositi, 1998) to infer from the texts their meanings and contexts of use (Krippendorff, 2013, p. 24). Through this method, texts are brought back to a limited number of categories using explicit analytical decomposition, classification, and coding procedures (Weber, 1990).

Content classification employs inductive coding strategies. In other words, instead of coding the texts based on *a priori* classifications, the classification is adapted in the process through the reading and re-reading of the texts. Text interpretation is carried out following the principles and techniques of the hermeneutic approach to social research (Montesperelli, 1998), which aimed to identify the widespread and shared common-sense dimensions related to the world of asexuality.

The following section will present some results. For ease of reading, we will begin by describing online presentations and interactions to construct a typology of asexual people. We will then try to see how these profiles are distributed according to the main socio-demographic variables deduced from the profile analysis. The aim is not only to arrive at a more complete analysis of the phenomenon but also to describe all the analytical procedures that can be used in this type of approach.

## Results

### Following mainstream models: Emotional fragility and social pressures

When registering to the community, users are required to fill in a personal profile with socio-biographical information, including their gender, sexual orientation, age, place of residence and some considerations about themselves in terms of a brief presentation. These are generally followed by comments from other users. The resulting interactions shed a light on the various points of view on asexuality.

To delve deeper into the motives that lead people to self-identify as asexual, we examined the presentations in profiles, comments, and general interactions within the platform.

Through the analysis of this information, we discovered some of the motivations that lead users to identify themselves as “asexual”. We identified both individual factors, such as personality traits or emotional aspects connected to experiences that led them to voluntarily renounce sexual relations, and social and cultural factors, partly referring to social pressures connected to stigmatization and discrimination.

About the former, fragility-related experiences frame the choice of asexuality. According to the studies by Carrigan (2012), Foster and Scherrer (2014) and Yule et al. (2015), low self-esteem and a lack of trust in others are positively correlated with the decision to renounce sexuality. This same issue is also highlighted in some posts, such as the one below.

*I am asexual. That is, I've never had sex and I don't care. The truth is that since childhood I have always been shy and awkward. My mother was alone, I mean I never knew my father and I have no brothers or sisters. It was always just the two of us, alone. In high school, I had few friends and those few had more problems than me. I currently study literature (...) and live in a house with other people. I have exchanged very few words with them. My life is full of silence and time. Perhaps I am asexual by choice, not my own. I see the future with fear (ID76)* ^3^

Asexuality is experienced with great difficulty because the obligation to sexuality is taken for granted in intimate relationships, often leading to the need to envisage strategies to avoid all situations where the pressure becomes stronger.

*Basically, I am absolutely not interested in sex, so much so that every time I fell in love I did absolutely nothing, I enjoyed being in their company every free moment, but constantly feared the moment when it would be inevitable to touch the subject (of course the “sexuals” expect it and at some point, sometimes pretending to joke about it, they will ask “but don't you like me? how come we never.. ?”)*

*Aware of being unfit to sustain a normal relationship, I have avoided it, I have tried to live with them exciting moments (and there have always been many), and I have carefully avoided situations in which we could risk finding ourselves alone in non-public places, I have always made sure that “it was getting so late that at that point I could at most offer them a ride home... like, you know, tomorrow I have a very busy day at work” (ID51)*.

Many users name social expectations and other people's pressures on their personal experiences as one of the main reasons which have, over time, dulled and in many cases extinguished their interest in sexuality and sexual practices. Self-presentations often reveal cases of marginalization, loneliness, and high demands in the life contexts of individuals, starting with the family and ending with social and/or educational contexts.

*I am terrible at introducing myself, so I'll get straight to the point. In my life, I have always perceived that something about me was different from my peers, until a few months ago. I was seen as the odd one out, maybe gay or who knows what was on his mind, perpetually lonely and not participating in male banter. As I grew up, I developed the “ability” to adapt and hide from others to make that awkwardness go away, which didn't belong to me, actually, since it had always seemed more like someone else's problem. I tried to be with girls, but they expected too much from me compared to what I could give both sexually and emotionally. The hardest thing was being able to talk and explain how I felt but when I tried to do that... “go to a psychologist and solve your problems”. Not that it helped much, actually, and the ironic thing, after all this time, at 35, I felt better watching BoJack Horseman and its explanation of Todd. Immediately afterwards I started to feel at peace with myself. A cartoon explained what I felt about myself and that above all I am not alone (ID80)*

*I am not exactly in my prime: I am 57 years old and for a long time now I have been, as I understand it, asexual. Sex has never been important to me. But I must say that since I got rid of it, I've been living much better. It was always a “gold rush” and many women made me feel inadequate because I was never good enough. Interest has steadily waned. Now I live my time with friends and people who have the same interests. Maybe I can find new friends even in this chat room. (ID123)*

Ethnographic observation of self-presentations and interactions shows that some users experience a condition that in some ways overlaps with voluntary social self-isolation, better known as the Hikikomori Effect.

Recent studies (Masullo, 2021) have shown that the phenomenon of voluntary social self-isolation is rapidly expanding, particularly in younger people, and that this phenomenon has redefined and reworked many processes of socialization. In this case, the redefinition also encompasses sexual aspects, as highlighted by this post:

*I hope I am on the right forum (...) I don't know if I can call myself asexual, but my situation is this. I have never had a relationship with anyone, and I have no sexual interest in anyone. I don't think I even know if I like boys or girls. But that's simply because I don't live among people, I haven't left the house since graduation. Yes, maybe I've gone out a few times to buy clothes or accompany my mother somewhere, but I tend to never leave the house and I don't have any friends, at least, not in Bergamo. So, I would like to know if there are people in this forum who are in the same situation as me? (ID44)*

### A typology of asexual people

The self-presentations and interactions on the platform allowed us to trace some of the specificities of people who define themselves as asexual or who are questioning their sexual identity, thus making it possible to obtain more details regarding the meaning of this choice, of which users are often not even clearly aware. The posts highlight two main characteristics, which would seem to ground or delineate certain ways of experiencing their condition as an asexual person, (even in the absence of shared definitions in the mainstream LGBTQ+ community): in simple terms, “love”, and sex. The former is how users consider and feel about the need to form relationships with others in sentimental terms, i.e., to build a meaningful relationship which can be a prelude to love and an emotionally fulfilling relationship. The latter is the degree of importance they attribute to sexual practices, which calls into play the need to relate with the other in a physical sense, in response to both a self-directed impulse (to feel sexual desire) and a hetero-directed one (in response to a social expectation connected to the influence of the main agencies of socialization to sexuality, including partners, family, friends, etc.,).

**Figure 1:**
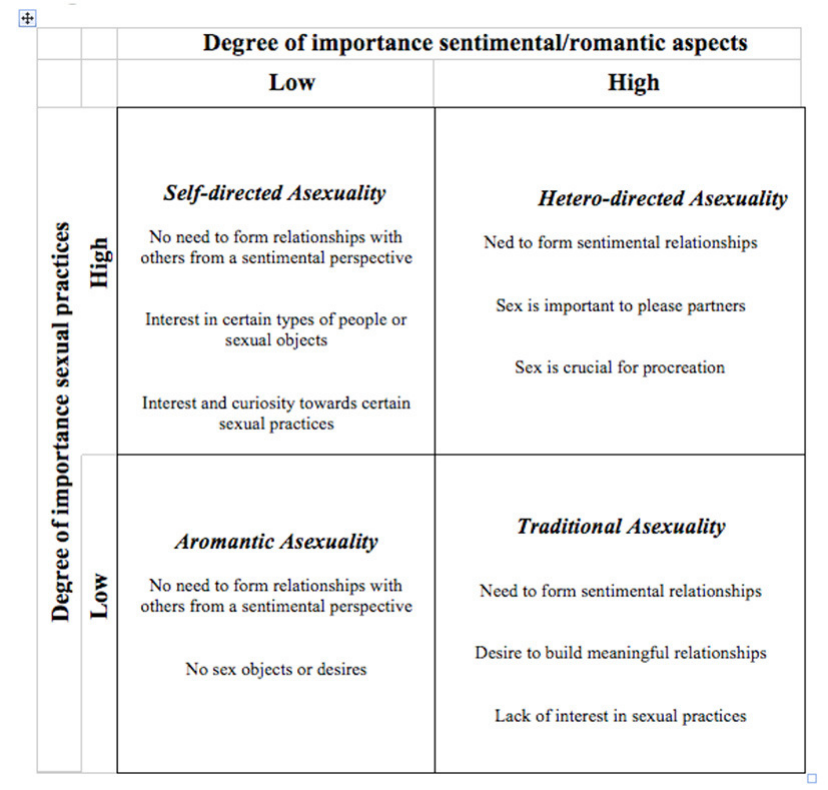
Typologies of users of Asexuality community.

Based on these dimensions, we constructed a typology of asexual identities, taking full advantage of the potential of digital ethnographic research (Kozinets, 2015; Masullo et al., 2020). The typology results from the intersection of two dimensions: the degree of importance attached to the construction of a romantic relationship and the degree of importance attached to sex. Four hypothetical ways of being an asexual person are thus highlighted, which can be summarized in the following diagram (Figure 1):

The first quadrant in the upper left-hand corner includes those in an initial process of self-reflection regarding their sexual identity, who also use the web community to gather information to better define themselves. We called them “self-directed” asexuals. This group comprises individuals who focus their attention on *sexuality per se* rather than on the need to build meaningful relationships with others in a sentimental or romantic sense. Their posts—often in the form of a question—focus on the meaning of sex *per se* and on certain sexual practices, toward which they express curiosity or, in some cases, aversion. In this case, we see a hybrid form of asexuality, not yet clarified, or transitory, as is shown in the following post:

*The aspect of autoeroticism and fantasies has not changed since I stopped seeking sexual relationships with partners. When I chat with someone, I try to sabotage any in-person meeting, I prefer to just have fantasies about that person, I think it is the right compromise between pleasure and self-protection (ID189)*

*I have read that many do not even practice masturbation, I couldn't do without it! But then I don't know if I can call myself asexual... is there someone like me who has no interest in sex with other people but doesn't stop pleasuring himself? (ID166)*

In the second box at the top right, we find “hetero-directed” asexuals, i.e., individuals who feel the need to build meaningful romantic relationships with others but, at the same time, feel no interest toward sex and, in general, all kinds of sexual practices. It is worth noticing that, in this group, sex does not disappear but takes on significance depending on the pressures exerted by the social environment. These may relate, for example, to the demands of a partner, the desire to conform socially to others or the need for sexuality to ensure the continuation of the species, as the following two excerpts show:

*Hello, in my life I have never felt a need to have sex, although I have made an effort to look like others, I am interested in sexual energies, (...) I am looking for simple acquaintance, on a friendship level and given my age I need “companionship”. I have many interests, I do meditation, I like traveling, and I like archaeology. (ID33)*

*Hi, I am an asexual guy who finds it unpleasant to have sex with girls and boys*.

*I think sex is not fundamental to a relationship but only a necessary act for reproduction. If you feel the same way and want to compare notes, you can write to me (ID38)*

The third box on the lower left is characterized by an exacerbation of relational closure toward others, a condition defined in the literature as AroACE or *asexual aromantics*. AroACE people are interested neither in the sexual aspects of the relationship nor in emotional and sentimental involvement.

*Aromantic Asexual, I love art, old films, reading, and sport. I love walking surrounded by nature. I like meeting new people, and establishing friendly and sharing relationships, like many of you I do not feel the need for a sexual relationship (ID20)*.

*I can define myself as asexual but also aromantic. Romantic love is only a concept, and a very recent one at that, just think of history, who married for love? Personally, I find it bothersome to think about sex and I find it hard to think about love. Friendship is already demanding enough. (ID6)*.

In the last box on the bottom right are the cases identified in the literature through the acronym ACE (Bogaert and Skorska, 2011; McInroy et al., 2021). They lack sexual impulses and a consequent reduction of sexual relations but maintain a strong desire to form relationships [with others] in romantic terms, according to the classic scheme of “platonic love”. This condition adheres to the purist conception of asexuality that is transversally evident in almost all users, without distinction for sexual orientation (that is, among both homosexual and heterosexual people). This is in line with recent theories that consider asexuality to be outside the official classification and taxonomies of sexual orientations. Below are two examples of typical presentations of ACE persons:

*I discovered I was asexual last January after I made a recap of all my relational experiences with both girls and boys, which were characterized by a total lack of sexual attraction (always on my part), but by romantic attraction with strong emotional ties; but, alas, I was rejected because they saw me more as a friend. Forgive me if I have not written much, I am a man of few words. (ID3)*.

*Good evening, everyone. (...) I'm asexual and have recently been living this condition of mine with serenity. I must say that in my youth I was ashamed, especially in groups or at home, I felt like I was wrong. But now I am happy that I don't have to hide. I like being in company, I love the mountains and traveling. But above all I like polite people, if I were to meet someone interesting, I'm open to a sentimental relationship. Of course, I am looking for an asexual partner (ID40)*

As per the research design, the final stage of the analysis, corresponding to the “reading” procedure, addressed the distribution of certain socio-biographic traits on the profiles of asexual persons on the platform. We reconstructed the latter through the typology presented above and deduced the former from the analysis of the profiles selected in the asexual community. Although we are aware that these data are not representative of the universe examined, we synthesized them from a statistical point of view, intending to also verify the relationships between the socio-biographical traits collected and certain characteristics associated in the literature with asexual people.

On the distribution of gender identity and sexual orientation on the profiles sampled, 68% of users self-identify with a male gender identity and 20% with a female gender identity. It is worth noticing that there is a significant presence of people declaring a non-binary gender identity (about 12%). The same applies to sexual orientation: while the majority identify as heterosexual (73.5%) or homosexual (about 17%), there is no shortage of people identifying as non-binary or pansexual (Table 1).

**Table 1:** Distribution of the sample surveyed according to gender and sexual orientation.

	**General cases**	**Gender M**	**Gender F**	**Another gender**	**Heterosexual**	**Homosexual**	**Other sexual orientation**
Total number of general cases	200	68%	20%	12%	73.5%	17%	12.5%

This difference in the incidence of males over females could lend itself to multiple interpretations, depending also on the different ways in which Italian men and women relate to sexuality and the most widespread collective imaginaries connected to it (Corbisiero and Nocenzi, 2022). While it is true that the current hypersexualisation of society affects all genders indiscriminately, prescribing a sort of “obligatory” sexuality, this takes on different meanings in the sample examined, also due to the different socialization paths to gender and sexuality experienced by men and women (Masullo, 2021). For the former, sexuality is a core aspect of the acquisition of a “hegemonic” male gender identity—thus a compulsory step as proof of one's appropriateness in the execution of one's gender role. For the latter, instead, sexuality is characterized by a lesser “centrality” which, even in the online environment, still fails to find an adequate space of explicitness (Masullo, 2021). In the case of men, the absence of sexual desire can be experienced as a source of concern, not least because of social pressures. For women, this condition is less felt, as sexuality remains relegated to the idea of a stable relationship and in specific cultural environments still bound to the idea of reproduction. In this sense, the absence of sexual desire is experienced by women with less concern, as this condition is regarded as a normal aspect of the process of socialization to sexuality, and subordinate to the need to establish an emotionally satisfying relationship.

All the users in the community define themselves as asexual or question their sexuality as falling into this category Out of the 200 profiles surveyed during the period under consideration, about 54% identify themselves as “tout court” asexuals, what we called “traditional asexuality” (ACE in the literature). They are characterized by the total or partial absence of sexual desire but wish to establish an emotional and sentimental relationship (Table 2). 32.5% of the profiles report total closure toward the other, lacking desire toward both sex and the need to establish a sentimental relationship, a condition defined as “Aromatic Asexual” (AroAce in the literature). Finally, 13.5 per cent of the users are unclear or uncertain about their identity: they show interest in sexuality or at least curiosity about it. This is true in both a hetero-directed and a self-directed sense. In the former case, for example, to accommodate the desires of a partner; in the latter, there is desire to experience certain practices such as sexting or cybersex). These people only partly fall within the category of asexuality—which, however, it is worth remembering, does not constitute a fixed identity but is subject to change and negotiation processes over time and in the spaces of online and offline sociality. Any attempt at classification would, therefore, prove inadequate, even if it is analytically valid when constructed to describe the phenomenon (Table 2).

**Table 2:** Distribution of the types of asexual persons.

**Asexual categories**	**Cases**	**%**
Traditional asexual	108	54
Aromantic asexual	65	32.5
Hetero-directed asexual	17	8.5
Self-directed asexual	10	5
Total	200	100

Finally, we explore the distribution of the identified types in class age following the criterion of division defined by the ISTAT and longitudinal surveys on youth which in the Italian context consider to be young people between 18 and 34 years old (Toniolo, 2022 and previous annual reports). Those under 18 years old may be considered teenager and those above 35 adults.

By cross-referencing the reconstructed categories of asexual people with the age groups considered, we can highlight the generational distribution of the phenomenon examined, as shown in Table 3.

**Table 3:** Distribution by age cohorts of the types of asexual people.

	**% Under 17**	**% 18–34**	**% 35–54**	**% Over 55**	**% Total**
Traditional asexual	42%	68%	26%	25%	54%
Aromantic asexual	40%	13%	59%	75%	32.5%
Hetero-directed asexual	4%	10%	10%	0	8.5%
self-directed asexual	14%	9%	5%	0	5%
Total 200 (v.a)	100% (50)	100% (107)	100% (42)	100% (4)	100% (203)

Traditional Asexuals (ACE in the literature) are mainly those between 18 and 34 years old (68%) and under 17 years old (42%). For the younger age cohorts, sentimental aspects seem to be more important than for the later cohorts. This is also evident if one compares this with the Aromatic Asexual condition (AroACE in the literature) which is more concentrated among the over 55-year-old (75%) followed by the 35–54 year-old (59%). It can be hypothesized that for the latter cohorts, the condition of aromantic asexuality is the outcome of a progressive disinterest in sexuality following unsatisfactory experiences.

The condition of aromatic asexuality is also evident among the under 17-year-old group (40%). It could be a “comfort choice” to delay the creation of sentimental and sexual relationships to avoid disappointment, alleviate relational performance anxiety, or for subjective reasons that would require a more in-depth study with qualitative research approaches.

The more complexly defined “self-directed” and “hetero-directed” asexual profiles refer to hybrid conditions ranging from situations which express curiosity only for certain sexual practices (such as sexting or cybersex) to others marked by a total lack of interest in sexuality, which is practiced only under external pressures.

They are more common among the teenagers (under 17-year-old) and the young people between 18 and 34 year-old compared to other cohorts.

Although it is not the purpose of this article to analyse the characteristics associated with these age cohorts, the greater propensity toward hybrid profiles of the younger generations (meaning both those under 17 and those between 18 and 34 years of age) could be associated with the complex and nuanced stage of their life with multiple sexual and romantic attractions, which are rarely static but fluctuate throughout their lives (Porrovecchio, 2012; Savin-Williams, 2021). Although the data would need further investigation, it can be hypothesized that the greater propensity of these age cohorts to experiment could indicate that the choice of asexuality is only temporary, or linked to specific relationships, and it can hardly be framed within the asexual condition tout court as defined in the literature.

## Discussion and conclusions

### Limitations and potential of digital ethnographic research and considerations on its application to the field of sexuality studies

The present study addressed asexual people and the processes of self-definition in the digital environment. The digital ethnographic research approach allowed us to explore some of the essential steps of the research design inspired by digital ethnography, highlighting the main techniques to employ, the dilemmas to resolve before commencing the fieldwork, and the types of analysis to carry out. The transposition of classical ethnographic techniques into the digital environment constitutes a resource for researchers who intend to explore phenomena concerning populations that are difficult to reach. Digital ethnography proves particularly suitable where such populations take on their specific connotation in digital spaces, as in the case considered here. Our research shows that, in the absence of a shared interpretation of asexuality, its definition results from the interaction with others, an intersubjective process occurring mainly in the digital environment and which has no place elsewhere. By offering the possibility of creating profiles, introducing and describing oneself, and commenting, the web community provides useful tools to arrive at a shared definition, create languages and socialize with them, and attribute meanings—the scripts of a digital subculture still in the making but with its specificities compared to others that make up the variegated LGBTQ+ universe. The study of the profiles, self-presentations and comments allowed us to explore how the asexual condition goes far beyond the question of sexual orientation, resulting instead from how people relate to a norm that sees sexuality as a “compulsory” step in the processes of gender and sex identification. It is no coincidence that in the web community examined, most of the users are men. For them, sex is the benchmark against which gender identity is socially tested. Asexuality can be seen as an indicator of a crisis of masculinity, a hypothesis that deserves future exploration with the help of other techniques and a larger sample.

The proposed typology of asexual persons highlights how the choice of asexuality is a process characterized by numerous ways of understanding sexuality and the desire for romantic relationships, marked by discontinuities more than endpoints. These subjective propensities depend on biographical, social, and imaginary experiences rather than natural predispositions or simplistic and essentialist readings of sexual identity. While this approach has its advantages, as highlighted by this case study, it is precisely within its framework that the concrete limitations to its application become apparent. The first refers to ethical issues, which directly call into question the role of the researcher and his positioning in the research field and the consequences produced by his representations. In the field of sexuality, in particular, critical approaches—such as postcolonial theory, feminist critique, and queer theory—have greatly emphasized the researcher as an interpreter of the Other/s, as a privileged observer who risks subordinating those being observed and described. The emphasis on reflexivity in social research makes it possible to understand how “meanings result from the interpretive negotiation occurring on the field between researchers and participating subjects as embedded subjects and producers of knowledge whose interactions (both in the field and through textual strategies) are filtered and constructed based on gender, sexuality, nationality, race and ethnicity, social class, age, and bodily ability” (Grassi et al., 2020, p. 111). Guided by these concerns, we chose to declare ourselves as researchers to deepen some reflections stemming from what we observed and to respect the point of view of the natives as much as possible. We were, indeed, well aware that the readings produced without this confrontation could unleash multiple consequences on subjects who are already vulnerable, and therefore expose them to further processes of marginalization and exclusion. A further issue of digital ethnography is taking into account the peculiarities of an observation that takes place in a digital environment. We must consider the effect of the medium of communication which, unlike in the case of face-to-face observation, often does not allow researchers to go deeper into the characteristics of the subjects. By conducting in-depth research through “questioning” (that is, privately contacting some users and asking them for details and clarifications), we intended to make up for some of these gaps. The discontinuity, nevertheless, remains “unbridgeable” compared to traditional face-to-face ethnographic research—the main limitation to be taken into account when choosing this type of approach. The future of digital ethnographic research will thus lie in the way it manages to meet some of these challenges, which will depend both on the researchers' ability to combine different research techniques, and on the technological evolution of the tools proposed by the web society. The latter seems to be increasingly moving toward overcoming the differences between real and virtual, between research carried face-to-face or remotely.

## Data availability statement

The original contributions presented in the study are included in the article/supplementary material, further inquiries can be directed to the corresponding author.

## Ethics statement

Ethical review and approval was not required for the study on human participants in accordance with the local legislation and institutional requirements. Written informed consent from the participants' legal guardian/next of kin was not required to participate in this study in accordance with the national legislation and the institutional requirements.

## Author contributions

The whole research is a result of intense collaboration among the authors. In the final draft, GM wrote sections: Introduction, The digital ethnographic approach: Similarities and differences with the traditional ethnographic approach, Research design, and A typology of asexual people. MC wrote sections: The phenomenon of asexuality: A literature review and Following mainstream models: Emotional fragility and social pressures. The authors co-wrote section Discussion and conclusions. Both authors contributed to the article and approved the submitted version.

## Footnotes

^1^Among the many definitions, we find: Cyber Ethnography (Morton, 2001), Ethnography of Virtual Spaces (Burrel, 2009), Virtual Ethnography (Hine, 2008), Internet Ethnography (Boyd, 2008), Ethnography on the Internet (Beaulieu, 2004), Internet-related ethnography (Postill and Pink, 2012); Webnography (Puri, 2007).

^2^While some considerations and results are akin to those emerged from the previous research on the AVEN community (Delli Paoli and Masullo, 2022), we will not be making any comparison. The phenomenon of asexuality is here a case study to present the digital ethnographic approach and not the aim of the essay.

^3^In order to preserve partecipants'anonimity we have used an alphanumerical code for each partecipant (ID^*^). For the same reason, we have dropped out any socio-biographical information.
